# F1 and Tbilisi Are Closely Related Brucellaphages Exhibiting Some Distinct Nucleotide Variations Which Determine the Host Specificity

**DOI:** 10.1128/genomeA.01250-13

**Published:** 2014-01-30

**Authors:** Jens A. Hammerl, Sascha Al Dahouk, Karsten Nöckler, Cornelia Göllner, Bernd Appel, Stefan Hertwig

**Affiliations:** Federal Institute for Risk Assessment (Bundesinstitut für Risikobewertung), Berlin, Germany

## Abstract

We report on the 41,143-bp genome of brucellaphage F1, a podovirus that infects several *Brucella* species. The F1 genome is almost identical to the genome of brucellaphage Tb. However, some structural proteins of the phages exhibit extensive polymorphisms and might be responsible for their different host ranges.

## GENOME ANNOUNCEMENT

Brucellae are facultative intracellular pathogens that may cause reproductive failure and abortion in animals and a feverish multiorgan disease in humans (1). Brucellaphages have been used as a diagnostic tool since the 1960s, when phage Tbilisi (Tb) was discovered (2). According to their host specificities, seven *Brucella* phage groups (prototypes Tb, Fi, Wb, Bk2, R/C, Iz, and Np) (3, 4) have been established, even though various host ranges have been suggested for phage Tb (3–5). The currently known brucellaphages share a podoviral morphology (6) and strong DNA homologies (4). However, only two brucellaphages (Tb and Pr) have been sequenced so far (7).

Here, we report the complete nucleotide sequence of phage F1 (8), which is routinely used for *Brucella* typing. F1 phage stocks were provided by the World Organisation for Animal Health (OIE) Brucellosis Reference Center of the Veterinary Laboratories Agency (Addlestone, United Kingdom). The phages were propagated on the *Brucella abortus* vaccine strain S19 and purified according to standard procedures (9). Similar to the host range of Tb reported by Corbel (3), F1 was propagated on *B. abortus*, *Brucella suis* (bv1 and bv5), *Brucella neotomae*, and *Brucella microti* reference strains. The plaques formed by F1 were large (2 mm in diameter) and clear. Like other brucellaphages, F1 has the typical morphology of a podovirus. Phage DNA was isolated by proteinase K/SDS treatment of CsCl-purified particles as previously described (9). Whole-genome sequencing was performed with the 454 genome sequencer FLX Titanium system (Roche, Germany). Assembling of reads (Newbler Assembler version 2.6) yielded a single linear contig with a >30-fold sequence coverage. Gene prediction and annotation of the genome sequence were carried out using MyRAST (10–12).

Phage F1 has a linear double-stranded genome of 41,143 bp, with an average G+C content of 48.2%. Bal31 analyses revealed that the genome is circularly permuted. Fifty-eight putative genes and seven transcriptional terminators have been identified. A functional assignment was made for 17 gene products. F1 is very similar to the sequenced brucellaphages Tb and Pr, with nucleotide identities of 99% and 98%, respectively. Most of the predicted gene products are even 100% identical. However, some F1 products that are presumably involved in virion assembly (gp12, major head protein; gp15 and gp16, structural proteins; gp24, tail spike protein) show striking amino acid polymorphisms in relation to the corresponding Tb proteins. Notably, Flores et al. (7) reported that the sequenced Tb phage exclusively infects *B. abortus* strains. Thus, it is very likely that the diverging structural proteins of F1 and Tb account for the diverging host range of the phages. Nevertheless, the high rates of identity suggest that the hitherto-described brucellaphages are host range mutants originating from a common ancestor. Since only a small number of F1 products diverge from Tb and Pr, it should soon be possible to identify the amino acids that are important for host specificity. This work can be facilitated by sequencing of brucellaphages that belong to other host range groups.

### Nucleotide sequence accession number.

The genome of brucellaphage F1 is available under the Genbank accession no. HG428758.
